# Differential Effects of GLP-1 Receptor Agonists on Cancer Risk in Obesity: A Nationwide Analysis of 1.1 Million Patients

**DOI:** 10.3390/cancers17010078

**Published:** 2024-12-30

**Authors:** Shauna Levy, Abdallah Attia, Rami M. Elshazli, Ahmed Abdelmaksoud, Danielle Tatum, Hani Aiash, Eman A. Toraih

**Affiliations:** 1 Department of Surgery, Tulane University School of Medicine, New Orleans, LA 70112, USA; aattia@tulane.edu (A.A.); relshazli@tulane.edu (R.M.E.); dtatum@tulane.edu (D.T.); 2 Department of Biochemistry and Molecular Genetics, Faculty of Physical Therapy, Horus University-Egypt, New Damietta 34517, Egypt; 3 Department of Biological Sciences, Faculty of Science, New Mansoura University, New Mansoura City 35742, Egypt; 4 Department of Internal Medicine, University of California, Riverside, CA 92521, USA; ahmed.abdelmaksoud@medsch.ucr.edu; 5 Department of cardiovascular perfusion, Upstate Medical University, Syracuse, NY 13210, USA; aiashh@upstate.edu; 6 Genetics Unit, Department of Histology and Cell Biology, Faculty of Medicine, Suez Canal University, Ismailia 41522, Egypt

**Keywords:** semaglutide, liraglutide, dulaglutide, cancer-risk reduction, personalized medicine

## Abstract

**Background**: Glucagon-like peptide-1 receptor agonists (GLP-1RAs) have demonstrated significant efficacy in obesity treatment beyond their original development for type-2 diabetes management. This comprehensive study investigated the relationship between GLP-1RA use and cancer incidence in individuals with obesity across a 5-year follow-up period. **Methods**: We conducted a large-scale cohort study using the TriNetX US Collaborative Network database (2013–2023) examining adult patients with obesity. The study utilized propensity score matching to pair GLP-1RA-treated patients with controls (1:1) using the nearest neighbor method. Cancer incidence served as the primary outcome measure over the 5-year follow-up, with subgroup analyses considering individual GLP-1RA agents, patient sex, and BMI categories. **Results**: Analysis revealed significant cancer-risk reductions associated with GLP-1RA use across multiple cancer types compared to matched controls. Notable risk reductions were observed in gastrointestinal (HR 0.67, 95% CI 0.59–0.75), skin (HR 0.62, 95% CI 0.55–0.70), breast (HR 0.72, 95% CI 0.64–0.82), female genital (HR 0.61, 95% CI 0.53–0.71), prostate (HR 0.68, 95% CI 0.58–0.80), and lymphoid/hematopoietic cancers (HR 0.69, 95% CI 0.60–0.80). Semaglutide demonstrated superior protective effects, particularly in gastrointestinal cancers (HR 0.45, 95% CI 0.37–0.53). Conversely, liraglutide showed increased risks for thyroid (HR 1.70, 95% CI 1.03–2.82) and respiratory cancers (HR 1.62, 95% CI 1.13–2.32). **Conclusions**: This research provides compelling evidence for GLP-1RA’s potential role in cancer-risk reduction, with semaglutide showing particularly promising results. The differential effects observed among GLP-1RA agents emphasize the importance of personalized medicine approaches. These findings suggest significant implications for clinical practice and future research in both obesity management and cancer prevention.

## Simple Summary

Glucagon-like peptide-1 receptor agonists are medications widely used for treating obesity and diabetes. While their metabolic benefits are well established, their potential impact on cancer risk remains unclear. This nationwide study of over 1.1 million patients examined how these medications affect cancer risk in people with obesity. Our research revealed that these drugs, particularly semaglutide, significantly reduced the risk of various cancers, though effects varied by specific medication type. These findings suggest that these medications may offer dual benefits in treating obesity while potentially reducing cancer risk, opening new possibilities for preventive medicine and personalized treatment approaches.

## 1. Introduction

Glucagon-like peptide-1 receptor agonists (GLP-1RAs), initially developed for type-2 diabetes management, have emerged as powerful tools in obesity treatment [1,2]. Researchers are increasingly focusing on understanding the full spectrum of GLP-1RA effects, including their impact on cardiovascular health and cancer risk [3,4,5]. While the complete range of benefits these medications offer remains to be fully understood, it is well demonstrated that metabolic and bariatric surgery (MBS) for weight loss leads to significant reductions in various health risks such as cardiovascular disease, diabetes, and cancer. A recent investigation by Aminian et al. [6] showed that MBS was associated with a significantly lower incidence of obesity-associated cancers over a follow-up period of 6.1 years.

GLP-1RA agents regulate nutrient assimilation and energy homeostasis by enhancing insulin secretion, inhibiting gastric acid secretion, affecting gut motility, and inducing satiety [2,7]. The widespread expression of GLP-1 receptors in various tissues, including the gastrointestinal tract, pancreatic islets, thyroid gland, kidney, heart, lungs, and central nervous system [8,9], suggests a broader physiological role beyond metabolic regulation [10,11]. This distribution raises intriguing possibilities regarding both therapeutic and adverse effects of GLP-1RAs, particularly their impact on cancer risk and progression [12]. The well-established link between obesity and increased cancer risk presents a unique opportunity to explore the potential dual benefit of GLP-1RAs in weight management and cancer-risk reduction [13].

Existing research into the relationship between GLP-1RA use and cancer risk has been limited by small sample sizes, short follow-up periods, or narrow focus on specific cancer types, and investigations have often yielded conflicting results. [14,15]. The FDA Adverse Event Reporting System (FAERS) reported a lower risk of prostate and lung cancer but a higher risk of thyroid cancer with GLP-1RA use [16,17]. Conversely, large cohort studies found no substantial increase in thyroid cancer risk over a mean follow-up of 3.9 years [18]. Given the enormity of the impact of cancer on the global population, there is an urgent need for comprehensive, large-scale studies examining the long-term effects of GLP-1RAs on cancer risk across multiple organ systems. Our study aimed to investigate the associations between various GLP-1RA types and cancer risk in a large cohort of individuals with obesity. We hypothesized that GLP-1RA use in this patient population would reduce cancer risk.

## 2. Materials and Methods

### 2.1. Study Design and Data Source

This was a retrospective cohort study analyzing longitudinal real-world data from the TriNetX US Collaborative Network. TriNetX is a federated research platform that aggregates de-identified electronic health records from 63 healthcare organizations across the United States [19]. The database contains comprehensive clinical information from over 93 million unique patients, capturing demographics, diagnoses (ICD-9/10, SNOMED), procedures (CPT, ICD), medications, laboratory results, vital signs, clinical measurements, healthcare encounters (inpatient, outpatient, emergency), and clinical observations. All data are standardized through consistent terminology mapping and validated through automated quality control processes.

Patients were selected between January 2013 and December 2023. The TriNetX platform enables real-time analyses while maintaining patient privacy through a federated architecture. To ensure data quality and standardization, we utilized comprehensive sets of diagnostic and procedural codes across multiple terminology systems and restricted our analysis to the US network to minimize coding practice variations across healthcare systems.

### 2.2. Study Population

The initial study population included adult patients (18–75 years old) with a body mass index (BMI) of 30 kg/m^2^ or greater (Table S1). Patients were excluded if they had any diagnosis of benign or malignant neoplasms prior to the index event, a history of bariatric surgery, treatment with discontinued incretin-based therapies (lixisenatide, albiglutide, exenatide), or pregnancy during the study period (Table S2).

**Figure 1:**
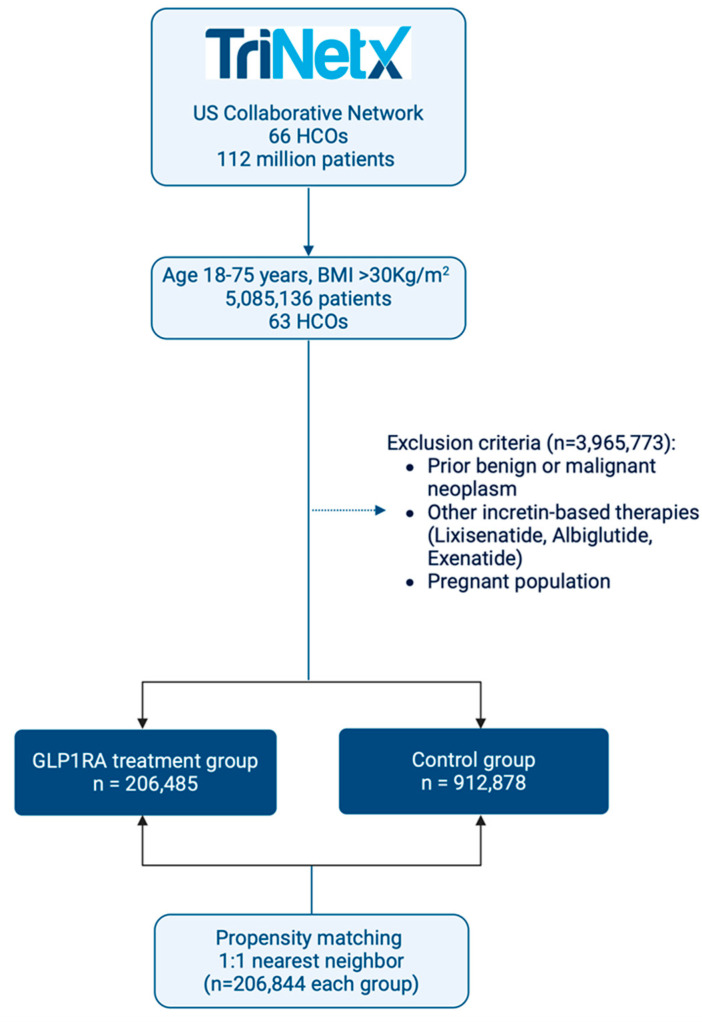
Flowchart representation of summarized study methods.

The treatment group included patients who met the same criteria and had received liraglutide, semaglutide, or dulaglutide, while the control group consisted of patients who did not receive these medications (Figure 1).

### 2.3. Outcomes

The primary outcome of interest was the incidence of malignant neoplasms, categorized by organ system and specific cancer types using ICD-10 codes (Table S3). Outcomes were assessed at 1-year, 3-year, and 5-year intervals post-index date of diagnosis or the start of GLP-1RAs treatment.

### 2.4. Subgroup Analysis

Stratified analyses by sex and BMI were conducted to identify similarities and differences in cancer risk. Additionally, drug-specific analyses that compared each GLP-1RA (semaglutide, liraglutide, dulaglutide) to its matched untreated cohort over the entire follow-up period were performed. Patients who switched treatments or received combination therapies were excluded from these analyses to maintain the integrity of drug-specific comparisons.

### 2.5. Propensity Score Matching

To minimize confounding and ensure comparability between treatment and control groups, propensity score matching was performed using the nearest-neighbor method with a 1:1 ratio and caliper width of 0.2 of the standard deviation of the logit of the propensity score. Propensity scores were calculated using a logistic regression model that incorporated comprehensive baseline characteristics using the triNetX built-in analytical tools. These included demographic factors (age at index, sex, race), clinical factors (baseline BMI, nicotine dependence [ICD10: F17, Z87.891], alcohol-use disorders [F10], history of irradiation [Z92.3], family history of cancer [Z80]), and key comorbidities (diabetes mellitus [E08–E13], and cardiovascular disease). After matching, we confirmed a balance between groups by comparing standardized differences, with values <0.1 considered well balanced.

### 2.6. Statistical Analysis

Descriptive statistics were used to summarize baseline characteristics. The risk of malignancy was compared between groups using relative risk (RR) and hazard ratios (HR) with 95% confidence intervals (95% CI). Subgroup analyses were performed to assess cancer risk stratified by specific GLP-1RAs and baseline BMI categories. Kaplan–Meier curves and competing risk analyses were used to evaluate outcome probabilities over time. Time-to-event analyses were conducted from the index date until cancer diagnosis, death, or end of follow-up. All analyses were performed using the TriNetX platform, with a two-sided *p*-value <0.05 considered statistically significant.

## 3. Results

### 3.1. Characteristics of Study Population

The study analyzed a large cohort of patients (n = 1,119,363) to examine the effects of GLP-1RA treatment on cancer risk in individuals with obesity. Prior to matching, there were significant differences between the treated group (n = 206,845) and the control group (n = 912,878) in baseline characteristics. The treated group had a slightly higher mean age (*p* < 0.001) and a higher proportion of females (*p* < 0.001). There were also statistically significant differences in racial composition and prevalence of comorbidities (*p* < 0.001 for all comparisons). After propensity score matching, the treated and control groups each included 206,844 patients with well-balanced baseline characteristics (Table 1).

### 3.2. GLP-1RA Use and Cancer Risk over Time

The incidence of malignant neoplasms among matched cohorts over the follow-up periods is provided in Table 2. Significant associations between GLP-1RA use and cancer risk across multiple organ systems over different time periods were observed (Table 3).

**Figure 2:**
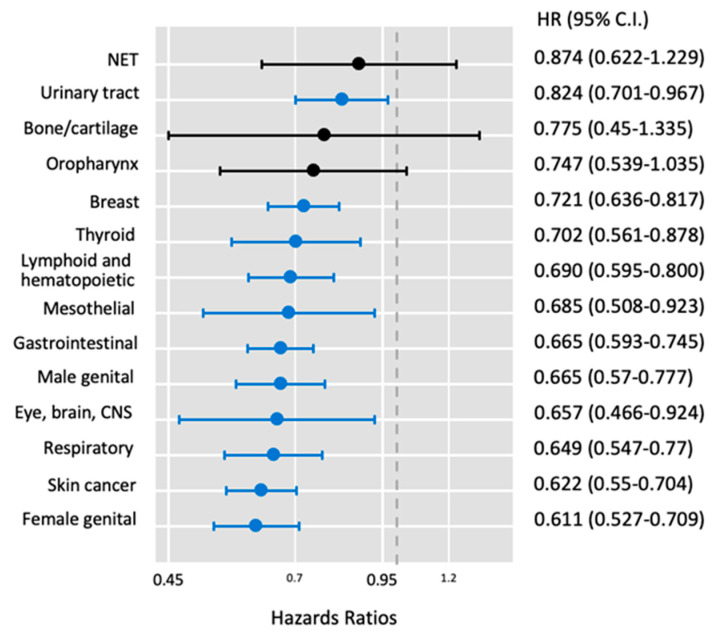
Five-year risk of malignant neoplasms. Cox regression analysis was performed in propensity-matched cohorts. Hazards ratio and 95% confidence interval (CI) are reported. Vertical dot line at HR of 1. Blue horizontal bars demonstrated significant protection against cancer in treated versus control groups. CNS: central nervous system, NET: malignant neuroendocrine tumors.

GLP-1RA use was associated with a protective effect on malignant neoplasms of digestive organs at 5-year follow-up (HR 0.66, 95% CI 0.59–0.75). This effect was particularly pronounced for colorectal cancer (HR 0.64, 95% CI 0.53–0.78) and liver/biliary cancers (HR 0.63, 95% CI 0.51–0.7). Female genital organ malignancies showed a lower association with GLP-1RA treatment at 5 years (HR 0.61, 95% CI 0.52–0.7), primarily driven by uterine cancer risk (HR 0.58, 95% CI 0.48–0.7). Similarly, breast cancer risk demonstrated lower cancer risk (HR 0.72, 95% CI 0.63–0.81). Male genital organ cancers also demonstrated reduced risk with GLP-1RA use at 5 years (HR 0.66, 95% CI 0.57–0.77), mainly due to decreased risk of prostate cancer (HR 0.68, 95% CI 0.58–0.79). Similarly, the risk of melanoma skin cancer was lower (HR 0.63, 95% CI 0.48–0.83). Other cancer types with significant risk reduction at 5 years included malignancies of the eye, brain, and central nervous system (HR 0.66, 95% CI 0.47–0.92) and thyroid and endocrine glands (HR 0.7, 95% CI 0.56–0.87). Notably, lymphoid and hematopoietic malignancies also showed a protective trend (HR 0.7, 95% CI 0.6–0.8), Figure 2.

### 3.3. Stratified Analysis of Cancer Risk by Sex

The sex-stratified analysis of GLP-1RA use and cancer risk revealed both shared and distinct patterns between males and females. The propensity-matched cohorts included 130,372 females and 76,875 males in both the treated and control groups, ensuring balanced baseline characteristics (Table 4). Both sexes exhibited significant risk reductions across multiple cancer types.

Gastrointestinal cancers showed marked decreases, with females experiencing a 31.3% risk reduction (HR = 0.69, 95% CI = 0.60–0.79) and males a 40.9% reduction (HR = 0.59, 95% CI = 0.51–0.68) (Table 5). Notably, colorectal and hepatobiliary cancers were significantly reduced in both sexes. Skin cancer risk decreased similarly, with females showing a 37.4% reduction (HR = 0.63, 95% CI = 0.53–0.74) and males a 39.5% reduction (HR = 0.61, 95% CI = 0.52–0.70). Both groups also experienced significant reductions in melanoma, thyroid, respiratory, and mesothelial cancers.

**Figure 3:**
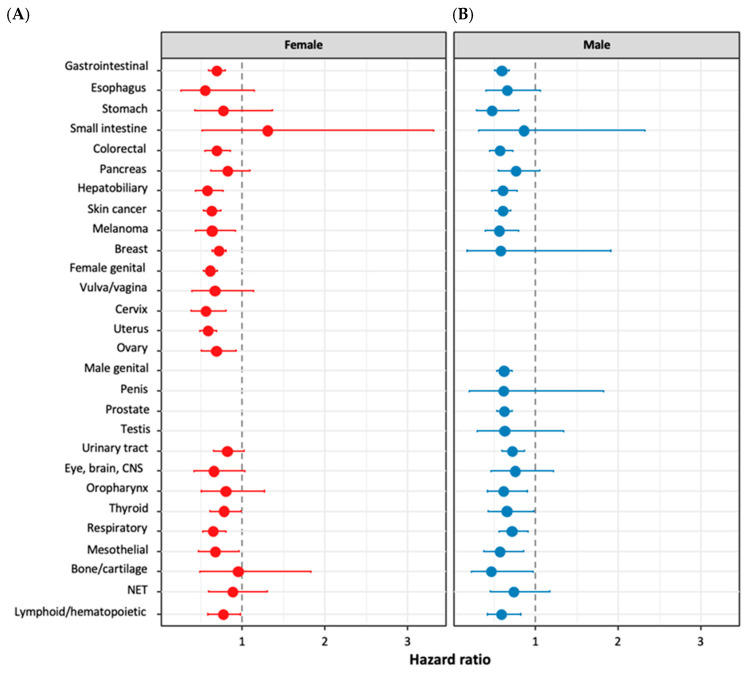
Sex-specific risk of cancer in patients treated with GLP-1R agonists. The magnitude of risk reduction in (**A**) female and (**B**) male patients treated with GLP-1R agonists compared to controls. Hazard ratios (HR) and 95% confidence intervals (CI) were calculated using Cox regression models. The forest plots illustrate the cancer-risk reduction across various organ systems, with HR less than 1 indicating a protective effect of GLP-1R agonist treatment. CNS: central nervous system, NET: malignant neuroendocrine tumors.

As depicted in Figure 3, sex-specific findings were also observed. Females demonstrated significant risk reductions in breast cancer (HR = 0.71, 95% CI = 0.63–0.8) and female genital organ cancers (HR = 0.61, 95% CI = 0.53–0.69), particularly cervical (HR = 0.55, 95% CI = 0.38–0.8), uterine (HR = 0.58, 95% CI = 0.49–0.7), and ovarian cancers (HR = 0.68, 95% CI = 0.51–0.92). In contrast, males exhibited significant risk reductions in stomach cancer (HR = 0.65, 95% CI = 0.28–0.8), prostate cancer (HR = 0.62, 95% CI = 0.53–0.72), urinary tract cancers (HR = 0.71, 95% CI = 0.6–0.87), and oropharyngeal cancers (HR = 0.61, 95% CI = 0.42–0.9). Interestingly, the protective effect against lymphoid and hematopoietic cancers was more pronounced in males (HR = 0.63, 95% CI = 0.52–0.77) compared to females (HR = 0.72, 95% CI = 0.6–0.87).

### 3.4. Subgroup Analysis of Cancer Risk by BMI

The BMI-stratified analysis revealed differential effects of GLP-1RAs on cancer risk between patients with BMI 30–39 kg/m^2^ and those with BMI ≥ 40 kg/m^2^ (Table 6). Both BMI groups showed significant risk reductions across multiple cancer types, but the magnitude of these reductions varied.

In the BMI 30–39 kg/m^2^ group, particularly strong protective effects were observed for gastrointestinal cancers, with notable reductions in colorectal and hepatobiliary cancers. This group also demonstrated substantial risk reductions in female genital cancers, particularly cervical and uterine cancers. The higher BMI group showed a more modest but still significant reduction in gastrointestinal cancer risk. Notably, this group demonstrated a significant reduction in pancreatic cancer risk, which was not observed in the lower BMI group. The protective effect on female genital cancers was less pronounced but still significant in this higher BMI group.

Both BMI groups showed similar protective effects against skin cancers, including melanoma. However, the risk reduction for breast cancer was more substantial in the lower BMI group (HR = 0.57, 95% CI = 0.50–0.65) compared to the higher BMI group (HR = 0.80, 95% CI = 0.67–0.96). Interestingly, the protective effect against thyroid cancer was more pronounced in the lower BMI group (HR = 0.46, 95% CI = 0.35–0.59) compared to the higher BMI group (HR = 0.68, 95% CI = 0.52–0.89). Both groups showed significant reductions in respiratory and lymphoid/hematopoietic cancer risks, with slightly stronger effects in the lower BMI group.

### 3.5. Subgroup Analysis of Cancer Risk by Individual Drugs

The assessment of cancer-risk profiles of different GLP-1RAs in individuals with obesity yielded varied results (Table 7). Semaglutide emerged as the standout performer in cancer-risk reduction. In a robust cohort of 100,494 matched pairs, it demonstrated a strong protective effect across a broad spectrum of cancer types. The gastrointestinal tract appeared to benefit most significantly, with risk reductions ranging from HR = 0.593 (95% CI = 0.407–0.864) for pancreatic cancer to HR = 0.328 (95% CI = 0.172–0.625) for stomach cancer. The protective effect of semaglutide extended beyond the digestive system, showing risk reductions in skin cancers (HR = 0.476, 95% CI = 0.393–0.576), breast cancer (HR = 0.465, 95% CI = 0.382–0.567), female genital cancers (HR = 0.387, 95% CI = 0.306–0.491), and male genital cancers (HR = 0.477, 95% CI = 0.371–0.615). Its impact on central nervous system cancers was also notable (HR = 0.257, 95% CI = 0.135–0.491).

The examination of liraglutide, which was an analysis of 28,629 matched pairs, revealed increased risks for several cancer types. Liraglutide was found to be associated with a significantly increased risk of mesothelial cancer (HR = 2.493, 95% CI = 1.309–4.746). Elevated risks were also observed for gastrointestinal (HR = 1.297, 95% CI = 1.037–1.622), breast (HR = 1.408, 95% CI = 1.099–1.803), thyroid (HR = 1.702, 95% CI = 1.029–2.815), respiratory (HR = 1.616, 95% CI = 1.127–2.318), and hematological cancers (HR = 1.347, 95% CI = 1.010–1.795).

Dulaglutide, examined in 42,717 matched pairs, presented an intermediate profile. It showed significant risk reductions in some cancers. Gastrointestinal cancers overall had a reduced risk (HR = 0.736, 95% CI = 0.605–0.896), with particularly strong effects on stomach (HR = 0.293, 95% CI = 0.135–0.637) and colorectal cancers (HR = 0.644, 95% CI = 0.469–0.884). Skin cancers were less frequent in dulaglutide users (HR = 0.585, 95% CI = 0.470–0.728), including a reduction in melanoma (HR = 0.513, 95% CI = 0.300–0.878). Female genital cancers showed a reduced risk (HR = 0.683, 95% CI = 0.521–0.895), primarily driven by a reduction in uterine cancer (HR = 0.688, 95% CI = 0.500–0.947). Male genital cancers had a lower risk (HR = 0.734, 95% CI = 0.573–0.940), particularly in prostate cancer (HR = 0.72, 95% CI = 0.558–0.928). However, dulaglutide did not show a significant impact on breast, thyroid, respiratory, or hematological cancers.

## 4. Discussion

This large-scale cohort study provides compelling support for the potential role of GLP-1RAs as a cancer-risk reduction agent in individuals with obesity. Our findings reveal a complex interplay between these drugs and cancer risk that extends beyond their established metabolic effects. Importantly, there was a consistent decrease in cancer risk across multiple organ systems over the five-year follow-up period. Collectively, our results demonstrated significant risk reductions encompassing 25 different cancer types, including digestive tract, skin (including melanoma), breast, and female genital organs, especially uterine cancer. Our study design provides unique methodological strengths through propensity score matching that balanced baseline BMI, diabetes status, and other relevant covariates between GLP-1RA users and controls. This matching approach helps isolate the effect of GLP-1RA treatment from confounding metabolic factors, suggesting potential protective effects independent of baseline weight status.

The association between GLP-1RAs and cancer risk remains a subject of debate. A meta-analysis by Hu et al. [20] investigated 45 clinical trials and found GLP-1RAs to have no significant effects on the occurrence of thyroid cancer. Our results showed that GLP-1RAs reduced the risk of thyroid and endocrine cancer. Sub-stratification by individual GLP-1RA type showed that semaglutide was associated with a significantly lower risk of thyroid cancer, while liraglutide increased that risk.

A 2020 study [15] meta-analysis concluded that GLP-1 treatment did not increase the relative risk of breast cancer. Similarly, GLP-1RAs were also demonstrated to exert an inhibitory effect on the growth of breast and cervical cancer, implying the potential application of GLP-1RAs for the treatment of these cancers [21]. Our results align with these previous findings, as there was a significantly lower 5-year risk of breast cancer. Our results further demonstrated that patients with a BMI between 30–40 kg/m^2^ had a significant risk reduction of developing breast cancer when compared to individuals with a BMI > 40 kg/m^2^.

Another meta-analysis conducted in 2022 [22] showed that liraglutide increased the risk of biliary cancers, while oral semaglutide decreased the risk. Our results agreed and demonstrated a similar increase in the risk of liver and biliary cancers with the use of liraglutide, while the use of semaglutide was associated with a significant risk reduction. Perhaps the most intriguing aspect of our findings was the marked difference in cancer-risk profiles among the individual GLP-1RA agents. Semaglutide demonstrated the strongest protective effects across a wide range of cancer types. Its efficacy in reducing gastrointestinal cancer risk was particularly strong for stomach cancer. The molecular basis for these effects likely involves multiple mechanisms. GLP-1 receptors are expressed on various cell types, making them direct targets for GLP-1RA action. These agents modulate key signaling pathways involved in cellular proliferation and survival, particularly through the ERK1/2 signaling cascade and the Notch and PI3K-AKT signaling pathways [23,24]. Furthermore, GLP-1RAs influence cellular methylation patterns critical for regulating gene expression related to cell growth and apoptosis [25] while also enhancing DNA repair mechanisms through increased expression of APE1, a key enzyme in base excision repair [26]. One potential explanation is that the unique pharmacokinetic profile of semaglutide, including its longer half-life and potentially enhanced tissue penetration, may contribute to its broader and more potent anti-cancer effects [8,27]. These findings align with preclinical studies showing the anti-proliferative and anti-inflammatory properties of semaglutide in various tissues [28,29].

Liraglutide exhibited a more complex risk profile, showing increased risks for certain cancer types, such as breast, thyroid, respiratory, and mesothelial cancers. These findings are noteworthy, considering liraglutide’s established safety profile in clinical trials [30,31]. Our findings regarding cancer risk with liraglutide use present a paradox in the current literature. While we observed increased risks for several cancer types, similar to other studies [32,33], these results contrast sharply with multiple meta-analyses and clinical trials that have found no significant increase in overall cancer or specific cancer risks [18,20,34]. This stark discrepancy between our large-scale observational study and previous clinical evidence underscores the complexity of assessing cancer risk in real-world settings. It suggests that despite their shared primary target, GLP-1RAs may have distinct off-target effects or tissue-specific impacts that differentially influence cancer risk. The increased risks observed in our study could be due to unmeasured confounding factors, differences in patient populations, or could represent true pharmacological effects that only become apparent in large-scale, long-term observational studies.

Dulaglutide presented an intermediate profile, with significant risk reductions observed for gastrointestinal, skin, female genital, and male genital cancers. However, the magnitude of these reductions was generally less pronounced than those seen with semaglutide. This pattern suggests that dulaglutide may offer some cancer-protective effects, but perhaps not to the same extent as semaglutide. This variability among GLP-1RAs challenges the notion of a class-wide effect and underscores the need for drug-specific evaluations in cancer-risk assessment. These findings are consistent with prior studies that found no increased risk of malignancy with dulaglutide use [34,35].

Our sex-stratified analysis revealed intriguing differences in cancer-risk reduction between males and females. While both sexes experienced significant risk reductions across multiple cancer types, the magnitude and specific cancer types affected varied. For instance, the more pronounced protective effect against lymphoid and hematopoietic cancers in males compared to females suggests potential interactions between GLP-1R signaling and sex-specific physiological factors. This observation aligns with the importance of considering sex-specific effects in pharmacological research and clinical decision-making [36,37].

The impact of BMI on cancer-risk reduction in our study reveals a complex relationship between obesity severity and GLP-1R agonists’ cancer-protective effects. While these drugs offer significant risk reduction across the obesity spectrum, their effectiveness varies with obesity degree and cancer type. The BMI 30–39 kg/m^2^ group showed stronger risk reductions for gastrointestinal and female genital cancers, suggesting greater efficacy in earlier obesity stages. Conversely, pancreatic cancer-risk reduction was significant only in the BMI ≥ 40 kg/m^2^ group, indicating different mechanisms in severe obesity. Breast and thyroid cancer-risk reductions also varied between BMI groups, supporting early intervention while suggesting sustained benefits even in advanced obesity. These patterns highlight the need for personalized cancer-risk reduction approaches in obese populations. Future research should explore the molecular and physiological basis of these BMI-dependent effects [1,38,39], potentially guiding the targeted use of GLP-1R agonists and new combination therapies across BMI categories.

Our observational findings suggest potential clinical considerations regarding GLP-1RA use in individuals with obesity, though these associations require validation through prospective clinical trials before directly informing clinical practice. In patients with elevated cancer risk, particularly gastrointestinal malignancies, semaglutide’s protective associations may warrant further investigation beyond its established metabolic effects. However, the increased risks observed with liraglutide for certain cancer types suggest the need for careful risk–benefit assessment. Dulaglutide’s intermediate risk profile could represent a balanced option for patients with moderate cancer-risk factors. These patterns highlight the importance of considering individual patient characteristics, including BMI and specific cancer-risk profiles, while acknowledging the limitations of observational data.

While these observational findings reveal intriguing associations between GLP-1RA use and cancer risk in individuals with obesity, several important limitations warrant consideration. As an epidemiologic study, it cannot definitively establish causality—only prospective randomized controlled trials can conclusively determine if GLP-1RA treatment directly influences cancer incidence. The notably superior cancer-preventing efficacy of semaglutide suggests the observed class-wide effects may be predominantly driven by its strong protective profile rather than representing a true class effect. While our rigorous propensity matching approach helps control for confounding, residual unmeasured factors and the inability to capture longitudinal weight changes limit our ability to distinguish between direct drug effects and weight loss-mediated benefits.

Future research directions should include prospective randomized controlled trials with longer follow-up periods, mechanistic studies investigating molecular differences among GLP-1RA types, and analyses differentiating between drug-specific and weight loss-mediated effects. Additionally, exploring potential synergistic effects with other cancer-risk reduction strategies could provide comprehensive approaches for cancer-risk management in obesity. These investigations would enhance our understanding of both the mechanisms of action and optimal clinical applications of GLP-1RAs in cancer-risk reduction.

## 5. Conclusions

Our study highlights the potential dual use of GLP-1RAs, not only as effective treatments for obesity and diabetes but also as promising agents for cancer-risk reduction. Semaglutide showed the most robust protective effects, while the profile of liraglutide indicated increased risks for certain cancers. These findings underscore the innovative potential of GLP-1RAs in oncology, offering new avenues for improving public health outcomes. Further research is warranted to elucidate the mechanisms and confirm the clinical benefits in reducing cancer risk.

## Figures and Tables

**Table 1:** Baseline characteristics of the study population before and after propensity score matching analysis.

Characteristics	Before Matching		*p*-Value	After Matching		*p*-Value
	Treated	Control		Treated	Control	
**Counts**	206,845	912,878		206,844	206,844	
**Age at Index**	**50.0 ± 13.9**	**49.7 ± 16.2**	**<0.001**	50.0 ± 13.9	50.0 ± 13.9	0.75
**Sex**						
Female	**130,374 (63)**	**554,297 (60.7)**	**<0.001**	130,373 (63)	130,237 (63)	0.66
Male	**76,422 (36.9)**	**358,309 (39.3)**		76,422 (36.9)	76,548 (37)	
**Race**						
White	**152,270 (73.6)**	**669,025 (73.3)**	**0.002**	152,269 (73.6)	152,386 (73.7)	0.68
Black or African American	**47,002 (22.7)**	**214,107 (23.5)**		47,002 (22.7)	46,903 (22.7)	
Asian	**4819 (2.3)**	**17,007 (1.9)**		4819 (2.3)	4840 (2.3)	
American Indian or Alaska Native	**800 (0.4)**	**3102 (0.3)**		800 (0.4)	670 (0.3)	
Native Hawaiian or Other Pacific Islander	**1954 (0.9)**	**9637 (1.1)**		1954 (0.9)	2045 (1)	
**Comorbidities**						
Nicotine dependence	**14,044 (6.8)**	**92,736 (10.2)**	**<0.001**	14044 (6.8)	14010 (6.8)	0.83
Alcohol-related disorders	**1720 (0.8)**	**17,114 (1.9)**	**<0.001**	1720 (0.8)	1702 (0.8)	0.76
History of irradiation	**200 (0.1)**	**1262 (0.1)**	**<0.001**	200 (0.1)	166 (0.1)	0.08
Diabetes mellitus	**105,883 (51.2)**	**227,210 (24.9)**	**<0.001**	105,882 (51.2)	105,930 (51.2)	0.881
Family history of cancer	**4390 (2.1)**	**21,379 (2.3)**	**<0.001**	4390 (2.1)	4348 (2.1)	0.65
Cardiovascular disease	**122,025 (59)**	**531,126 (58.2)**	**<0.001**	122,025 (59)	122,291 (59.1)	0.4

Data are presented as a number (percentage) or mean ± standard deviation. Two-sided chi-square or Student’s *t*-tests were used. Values denoted in bold font indicate statistical significance at a level of *p* < 0.05.

**Table 2:** Incidence of malignant neoplasms in propensity matched cohorts.

Cancer Type	Within 1 Year		*p*-Value	Within 3 Years		*p*-Value	Within 5 Years		*p*-Value
	Treated	Control		Control	Treated		Treated	Control	
**Gastrointestinal**	253 (12.2)	268 (13)	0.51	**420 (20.3)**	**521 (25.2)**	**0.001**	**550 (26.6)**	**648 (31.3)**	**0.005**
Esophagus	12 (0.6)	19 (0.9)	0.21	28 (1.4)	40 (1.9)	0.15	37 (1.8)	51 (2.5)	0.14
Stomach	11 (0.5)	18 (0.9)	0.19	29 (1.4)	38 (1.8)	0.27	37 (1.8)	52 (2.5)	0.11
Small intestine	10 (0.5)	10 (0.5)	1.00	11 (0.5)	10 (0.5)	0.83	17 (0.8)	10 (0.5)	0.18
Colorectal	101 (4.9)	111 (5.4)	0.49	**164 (7.9)**	**221 (10.7)**	**0.004**	**213 (10.3)**	**264 (12.8)**	**0.019**
Pancreas	71 (3.4)	57 (2.8)	0.22	109 (5.3)	109 (5.3)	1.00	142 (6.9)	138 (6.7)	0.81
Hepatobiliary	72 (3.5)	77 (3.7)	0.68	118 (5.7)	150 (7.3)	0.05	**157 (7.6)**	**195 (9.4)**	**0.043**
**Skin cancer**	**141 (6.8)**	**189 (9.1)**	**0.008**	**347 (16.8)**	**409 (19.8)**	**0.024**	**457 (22.1)**	**560 (27.1)**	**0.001**
Melanoma	41 (2)	36 (1.7)	0.57	77 (3.7)	74 (3.6)	0.81	92 (4.4)	112 (5.4)	0.16
**Breast**	237 (11.5)	207 (10)	0.15	386 (18.7)	410 (19.8)	0.40	475 (23)	518 (25)	0.17
**Female genital**	173 (8.4)	196 (9.5)	0.23	**255 (12.3)**	**330 (16)**	**0.002**	**309 (14.9)**	**404 (19.5)**	**<0.001**
Vulva/vagina	10 (0.5)	10 (0.5)	1.00	14 (0.7)	15 (0.7)	0.85	22 (1.1)	18 (0.9)	0.53
Cervix	**13 (0.6)**	**26 (1.3)**	**0.037**	34 (1.6)	39 (1.9)	0.56	41 (2)	44 (2.1)	0.75
Uterus	124 (6)	138 (6.7)	0.39	**170 (8.2)**	**229 (11.1)**	**0.003**	**203 (9.8)**	**279 (13.5)**	**0.001**
Ovary	40 (1.9)	38 (1.8)	0.82	59 (2.9)	73 (3.5)	0.22	72 (3.5)	91 (4.4)	0.14
**Male genital**	143 (6.9)	128 (6.2)	0.36	**224 (10.8)**	**280 (13.5)**	**0.013**	**296 (14.3)**	**346 (16.7)**	**0.048**
Penis	10 (0.5)	10 (0.5)	1.00	10 (0.5)	10 (0.5)	1.00	10 (0.5)	10 (0.5)	1.00
Prostate	136 (6.6)	112 (5.4)	0.13	**214 (10.3)**	**257 (12.4)**	**0.047**	283 (13.7)	323 (15.6)	0.10
Testis	10 (0.5)	11 (0.5)	0.83	10 (0.5)	17 (0.8)	0.18	10 (0.5)	17 (0.8)	0.18
**Urinary tract**	160 (7.7)	130 (6.3)	0.08	242 (11.7)	229 (11.1)	0.55	304 (14.7)	292 (14.1)	0.62
**Eye, brain, CNS**	28 (1.4)	33 (1.6)	0.52	48 (2.3)	65 (3.1)	0.11	60 (2.9)	73 (3.5)	0.26
**Oropharynx**	33 (1.6)	25 (1.2)	0.29	53 (2.6)	58 (2.8)	0.64	71 (3.4)	74 (3.6)	0.80
**Thyroid**	79 (3.8)	86 (4.2)	0.59	114 (5.5)	139 (6.7)	0.12	144 (7)	164 (7.9)	0.25
**Respiratory**	119 (5.8)	120 (5.8)	0.95	**183 (8.8)**	**234 (11.3)**	**0.012**	**240 (11.6)**	**290 (14)**	**0.03**
**Mesothelial**	34 (1.6)	46 (2.2)	0.18	61 (2.9)	76 (3.7)	0.20	81 (3.9)	93 (4.5)	0.36
**Bone/cartilage**	12 (0.6)	10 (0.5)	0.67	21 (1)	20 (1)	0.88	26 (1.3)	26 (1.3)	1.00
**NET**	34 (1.6)	30 (1.5)	0.62	61 (2.9)	49 (2.4)	0.25	70 (3.4)	63 (3)	0.54
**Lymphoid and hematopoietic**	150 (7.3)	170 (8.2)	0.26	262 (12.7)	298 (14.4)	0.13	330 (16)	375 (18.1)	0.09

Data are presented as a number (percentage). Two-sided chi-square test was used. CNS: central nervous system; NET: Malignant neuroendocrine tumors. Values denoted in bold font indicate statistical significance at a level of *p* < 0.05.

**Table 3:** Cancer-risk analysis in propensity-matched cohorts over 1-, 3-, and 5-year follow-up period.

Cancer Type	Within 1 Year		Within 3 Years		Within 5 Years	
	HR	95% CI	HR	95% CI	HR	95% CI
**Gastrointestinal**	**0.82**	**(0.690, 0.974)**	**0.66**	(0.580, 0.750)	**0.665**	**(0.593, 0.745)**
Esophagus	0.55	(0.267, 1.133)	**0.565**	(0.348, 0.916)	**0.56**	**(0.367, 0.856)**
Stomach	0.522	(0.246, 1.105)	**0.609**	(0.375, 0.988)	**0.543**	**(0.356, 0.828)**
Small intestine	1.467	(0.350, 6.141)	1.477	(0.546, 3.999)	1.286	(0.588, 2.812)
Colorectal	0.788	(0.602, 1.032)	**0.606**	(0.495, 0.741)	**0.633**	**(0.529, 0.759)**
Pancreas	1.086	(0.767, 1.540)	0.823	(0.631, 1.074)	0.811	(0.641, 1.025)
Hepatobiliary	0.811	(0.588, 1.118)	**0.644**	**(0.506, 0.819)**	**0.628**	**(0.508, 0.775)**
**Skin cancer**	**0.631**	**(0.507, 0.785)**	**0.677**	**(0.587, 0.782)**	**0.622**	**(0.550, 0.704)**
Melanoma	0.972	(0.621, 1.521)	0.837	(0.609, 1.152)	**0.631**	**(0.479, 0.832)**
**Breast**	0.989	(0.821, 1.192)	**0.77**	**(0.670, 0.885)**	**0.721**	**(0.636, 0.817)**
**Female genital**	**0.768**	**(0.626, 0.943)**	**0.641**	**(0.545, 0.755)**	**0.611**	**(0.527, 0.709)**
Vulva/vagina	1.324	(0.471, 3.721)	0.768	(0.371, 1.593)	0.956	(0.512, 1.784)
Cervix	**0.437**	**(0.225, 0.851)**	0.717	(0.453, 1.136)	0.745	(0.486, 1.140)
Uterus	**0.781**	**(0.613, 0.995)**	**0.617**	**(0.506, 0.752)**	**0.582**	**(0.486, 0.698)**
Ovary	0.914	(0.586, 1.425)	**0.668**	**(0.474, 0.941)**	**0.628**	**(0.461, 0.856)**
**Male genital**	0.959	(0.756, 1.218)	**0.648**	**(0.544, 0.773)**	**0.665**	**(0.570, 0.777)**
Penis	0.345	(0.067, 1.781)	0.417	(0.104, 1.668)	0.544	(0.153, 1.931)
Prostate	1.044	(0.813, 1.341)	**0.674**	**(0.562, 0.808)**	**0.68**	**(0.580, 0.798)**
Testis	0.383	(0.133, 1.102)	**0.382**	**(0.165, 0.886)**	0.47	(0.215, 1.028)
**Urinary tract**	1.07	(0.849, 1.349)	0.874	(0.729, 1.047)	**0.824**	**(0.701, 0.967)**
**Eye, brain, CNS**	0.74	(0.447, 1.225)	**0.607**	**(0.418, 0.882)**	**0.657**	**(0.466, 0.924)**
**Oropharynx**	1.145	(0.681, 1.926)	0.744	(0.512, 1.080)	0.747	(0.539, 1.035)
**Thyroid**	0.797	(0.587, 1.082)	**0.68**	**(0.531, 0.872)**	**0.702**	**(0.561, 0.878)**
**Respiratory**	0.855	(0.663, 1.102)	**0.64**	**(0.527, 0.777)**	**0.649**	**(0.547, 0.770)**
**Mesothelial**	**0.637**	**(0.409, 0.993)**	**0.657**	**(0.469, 0.921)**	**0.685**	**(0.508, 0.923)**
**Bone/cartilage**	1.13	(0.476, 2.683)	0.849	(0.460, 1.567)	0.775	(0.450, 1.335)
**NET**	0.966	(0.591, 1.579)	1.014	(0.696, 1.478)	0.874	(0.622, 1.229)
**Lymphoid/hematopoietic**	**0.765**	**(0.614, 0.953)**	**0.719**	**(0.609, 0.849)**	**0.69**	**(0.595, 0.800)**

CNS: central nervous system; NET: malignant neuroendocrine tumors; HR: hazard ratio; CI: confidence interval. Values denoted in bold font indicate statistical significance at a level of *p* < 0.05.

**Table 4:** Characteristics of propensity score matched cohorts stratified by sex.

Characteristics	Female		*p*-Value	Male		*p*-Value
	Treated	Control		Treated	Control	
Count	130,372	130,372		76,875	76,875	
**Demographics**						
Age at index	48.8 ± 14.1	48.8 ± 14.2	0.95	51.9 ± 13.2	52.0 ± 13.1	0.75
Race						
White	92,310 (70.8)	92,403 (70.9)	0.69	60,354 (78.5)	60,388 (78.6)	0.83
Black	33,907 (26)	33,814 (25.9)		13,103 (17)	13,127 (17.1)	
Asian	2656 (2)	2723 (2.1)		2166 (2.8)	2115 (2.8)	
Native Hawaiian	990 (0.8)	1040 (0.8)		290 (0.4)	236 (0.3)	
**Comorbidities**						
Smoking	7538 (5.8)	7522 (5.8)	0.89	6573 (8.6)	6596 (8.6)	0.83
Alcohol-related disorders	549 (0.4)	542 (0.4)	0.83	1179 (1.5)	1155 (1.5)	0.62
Diabetes mellitus	57,380 (44)	57,387 (44)	0.98	48,623 (63.2)	48,646 (63.3)	0.90
Cardiovascular disease	67,770 (52)	68,047 (52.2)	0.28	54,512 (70.9)	54,601 (71)	0.62
Personal history of irradiation	127 (0.1)	97 (0.1)	0.05	73 (0.1)	60 (0.1)	0.26
Family history of cancer	3180 (2.4)	3171 (2.4)	0.91	1215 (1.6)	1176 (1.5)	0.42

Data are presented as a number (percentage) or mean ± standard deviation. Two-sided chi-square or Student’s *t*-tests were used.

**Table 5:** Sex-stratified cancer risk analysis in GLP-1RA-treated versus untreated patients.

Cancer Type	Female		Male	
	HR	95%CI	HR	95%CI
**Gastrointestinal**	**0.687**	**(0.595, 0.793)**	**0.591**	**(0.511, 0.684)**
Esophagus	0.549	(0.262, 1.150)	0.656	(0.404, 1.065)
Stomach	0.765	(0.429, 1.364)	**0.476**	**(0.284, 0.797)**
Small intestine	1.302	(0.512, 3.311)	0.859	(0.317, 2.328)
Colorectal	**0.688**	**(0.552, 0.857)**	**0.566**	**(0.443, 0.723)**
Pancreas	0.822	(0.617, 1.096)	0.763	(0.556, 1.048)
Hepatobiliary	**0.578**	**(0.437, 0.764)**	**0.606**	**(0.473, 0.778)**
**Skin cancer**	**0.626**	**(0.529, 0.741)**	**0.605**	**(0.519, 0.704)**
Melanoma	**0.631**	**(0.434, 0.917)**	**0.561**	**(0.395, 0.797)**
**Breast**	**0.717**	**(0.638, 0.805)**	0.58	(0.176, 1.911)
**Female genital**	**0.608**	**(0.529, 0.699)**	-	-
Vulva/vagina	0.668	(0.391, 1.141)	-	-
Cervix	**0.554**	**(0.382, 0.803)**	-	-
Uterus	**0.581**	**(0.490, 0.690)**	-	-
Ovary	**0.684**	**(0.508, 0.922)**	-	-
**Male genital**	-	-	**0.618**	**(0.535, 0.715)**
Penis	-	-	0.611	(0.204, 1.829)
Prostate	-	-	**0.62**	**(0.534, 0.720)**
Testis	-	-	0.626	(0.293, 1.338)
**Urinary tract**	0.817	(0.651, 1.026)	**0.719**	**(0.593, 0.871)**
**Eye, brain, CNS**	0.652	(0.413, 1.028)	0.751	(0.465, 1.216)
**Oropharynx**	0.798	(0.502, 1.268)	**0.615**	**(0.421, 0.899)**
**Thyroid**	**0.775**	**(0.606, 0.991)**	**0.652**	**(0.431, 0.986)**
**Respiratory**	**0.648**	**(0.522, 0.805)**	**0.714**	**(0.561, 0.909)**
**Mesothelial**	**0.672**	**(0.471, 0.958)**	**0.571**	**(0.380, 0.859)**
**Bone/cartilage**	0.949	(0.491, 1.832)	**0.467**	**(0.224, 0.973)**
**NET**	0.881	(0.596, 1.300)	0.735	(0.459, 1.177)
**Lymphoid and hematopoietic**	**0.719**	**(0.595, 0.870)**	**0.628**	**(0.516, 0.765)**

Cox regression analysis was performed on propensity-matched cohorts of 130,372 females and 76,875 males in both treated and untreated groups. HR: hazard ratio; CI: confidence interval; CNS: central nervous system; NET: Malignant neuroendocrine tumors. Values denoted in bold font indicate statistical significance at a level of *p* < 0.05.

**Table 6:** Cancer-risk analysis in GLP-1RA-treated versus untreated patients stratified by BMI.

Cancer Type	BMI 30–39 kg/m^2^		BMI > 40 kg/m^2^	
	HR	95% CI	HR	95% CI
**Gastrointestinal**	**0.57**	**(0.509, 0.646)**	**0.65**	**(0.557, 0.757)**
Esophagus	0.63	(0.395, 1.018)	**0.42**	**(0.232, 0.763)**
Stomach	**0.58**	**(0.371, 0.906)**	**0.52**	**(0.288, 0.943)**
Small intestine	0.76	(0.359, 1.592)	1.61	(0.494, 5.227)
Colorectal	**0.51**	**(0.420, 0.614)**	**0.66**	**(0.518, 0.831)**
Pancreas	0.89	(0.695, 1.142)	**0.69**	**(0.498, 0.959)**
Hepatobiliary	**0.48**	**(0.389, 0.602)**	**0.73**	**(0.552, 0.974)**
**Skin cancer**	**0.54**	**(0.478, 0.614)**	**0.52**	**(0.435, 0.628)**
Melanoma	**0.64**	**(0.477, 0.858)**	**0.49**	**(0.321, 0.756)**
**Breast**	**0.57**	**(0.496, 0.649)**	**0.80**	**(0.668, 0.958)**
**Female genital**	**0.43**	**(0.360, 0.519)**	**0.64**	**(0.537, 0.768)**
Vulva/vagina	0.60	(0.334, 1.093)	0.95	(0.375, 2.412)
Cervix	**0.36**	**(0.227, 0.574)**	0.69	(0.425, 1.127)
Uterus	**0.38**	**(0.296, 0.482)**	**0.67**	**(0.539, 0.824)**
Ovary	**0.51**	**(0.363, 0.710)**	**0.53**	**(0.351, 0.806)**
**Male genital**	**0.56**	**(0.477, 0.660)**	**0.54**	**(0.420, 0.690)**
Penis	0.94	(0.249, 3.551)	0.30	(0.059, 1.567)
Prostate	**0.56**	**(0.473, 0.659)**	**0.54**	**(0.413, 0.692)**
Testis	0.47	(0.195, 1.150)	1.01	(0.319, 3.188)
**Urinary tract**	**0.67**	**(0.569, 0.800)**	**0.65**	**(0.524, 0.817)**
**Eye, brain, CNS**	**0.56**	**(0.376, 0.839)**	0.73	(0.455, 1.156)
**Oropharynx**	**0.52**	**(0.370, 0.741)**	**0.59**	**(0.387, 0.895)**
**Thyroid**	**0.46**	**(0.351, 0.589)**	**0.68**	**(0.518, 0.894)**
**Respiratory**	**0.52**	**(0.434, 0.619)**	**0.47**	**(0.365, 0.595)**
**Mesothelial**	**0.60**	**(0.432, 0.825)**	**0.65**	**(0.441, 0.966)**
**Bone/cartilage**	0.82	(0.452, 1.484)	0.64	(0.323, 1.273)
**NET**	**0.65**	**(0.459, 0.930)**	0.81	(0.526, 1.245)
**Lymphoid and hematopoietic**	**0.53**	**(0.450, 0.616)**	**0.60**	**(0.495, 0.733)**

Cox regression analysis was performed on propensity-matched cohorts. The BMI 30–39 kg/m^2^ group included 119,385 treated vs. 119,385 untreated patients. The BMI ≥ 40 kg/m^2^ group included 102,837 treated vs. 102,837 untreated patients. BMI: body mass index; HR: hazard ratio; CI: confidence interval; CNS: central nervous system; NET: malignant neuroendocrine tumors. Values denoted in bold font indicate statistical significance at a level of *p* < 0.05.

**Table 7:** Cancer risk stratified by individual drugs.

Cancer Type	Semaglutide		Liraglutide		Dulaglutide	
	HR	95% CI	HR	95% CI	HR	95% CI
**Gastrointestinal**	**0.446**	**(0.373, 0.534)**	**1.297**	**(1.037, 1.622)**	**0.736**	**(0.605, 0.896)**
Esophagus	**0.362**	**(0.178, 0.737)**	1.018	(0.434, 2.390)	0.598	(0.287, 1.246)
Stomach	**0.328**	**(0.172, 0.625)**	1.35	(0.600, 3.035)	**0.293**	**(0.135, 0.637)**
Small intestine	0.625	(0.217, 1.802)	2.873	(0.618, 13.358)	0.448	(0.100, 2.006)
Colorectal	**0.429**	**(0.327, 0.564)**	1.199	(0.846, 1.698)	**0.644**	**(0.469, 0.884)**
Pancreas	**0.593**	**(0.407, 0.864)**	1.416	(0.865, 2.317)	1.197	(0.804, 1.783)
Hepatobiliary	**0.46**	**(0.324, 0.653)**	1.453	(0.963, 2.193)	0.778	(0.543, 1.117)
**Skin cancer**	**0.476**	**(0.393, 0.576)**	1.151	(0.906, 1.462)	**0.585**	**(0.470, 0.728)**
Melanoma	**0.548**	**(0.367, 0.818)**	1.235	(0.691, 2.206)	**0.513**	**(0.300, 0.878)**
**Breast**	**0.465**	**(0.382, 0.567)**	**1.408**	**(1.099, 1.803)**	0.866	(0.672, 1.114)
**Female genital**	**0.387**	**(0.306, 0.491)**	0.964	(0.733, 1.268)	**0.683**	**(0.521, 0.895)**
Vulva/vagina	**0.293**	**(0.105, 0.814)**	0.838	(0.254, 2.767)	1.497	(0.560, 3.999)
Cervix	**0.3**	**(0.166, 0.542)**	1.233	(0.585, 2.601)	0.453	(0.203, 1.011)
Uterus	**0.326**	**(0.240, 0.442)**	0.954	(0.684, 1.331)	**0.688**	**(0.500, 0.947)**
Ovary	**0.544**	**(0.338, 0.875)**	1.047	(0.597, 1.836)	0.685	(0.345, 1.361)
**Male genital**	**0.477**	**(0.371, 0.615)**	1.184	(0.853, 1.642)	**0.734**	**(0.573, 0.940)**
Penis	-	-	0.725	(0.101, 5.211)	1.117	(0.186, 6.709)
Prostate	**0.483**	**(0.372, 0.626)**	1.184	(0.846, 1.657)	**0.72**	**(0.558, 0.928)**
Testis	0.688	(0.231, 2.050)	2.073	(0.214, 20.031)	1.062	(0.237, 4.764)
**Urinary tract**	**0.464**	**(0.366, 0.589)**	1.347	(0.983, 1.846)	0.782	(0.600, 1.020)
**Eye, brain, CNS**	**0.257**	**(0.135, 0.491)**	1.735	(0.858, 3.507)	1.382	(0.717, 2.661)
**Oropharynx**	**0.436**	**(0.257, 0.740)**	1.714	(0.936, 3.139)	**0.551**	**(0.312, 0.971)**
**Thyroid**	**0.543**	**(0.398, 0.740)**	**1.702**	**(1.029, 2.815)**	0.961	(0.604, 1.527)
**Respiratory**	**0.468**	**(0.349, 0.629)**	**1.616**	**(1.127, 2.318)**	0.896	(0.658, 1.221)
**Mesothelial**	**0.434**	**(0.259, 0.727)**	**2.493**	**(1.309, 4.746)**	0.694	(0.406, 1.185)
**Bone/cartilage**	0.471	(0.198, 1.124)	1.77	(0.684, 4.577)	0.474	(0.133, 1.684)
**NET**	**0.491**	**(0.290, 0.833)**	1.642	(0.876, 3.078)	1.173	(0.650, 2.118)
**Lymphoid/hematopoietic**	**0.382**	**(0.301, 0.485)**	**1.347**	**(1.010, 1.795)**	0.782	(0.606, 1.009)

CNS: central nervous system; NET: malignant neuroendocrine tumors; HR: hazard ratio; CI: confidence interval. Cox regression analysis was performed to identify cancer risk in drug users versus matched untreated cohort. Values denoted in bold font indicate statistical significance at a level of *p* < 0.05.

## Data Availability

The data presented in this study were obtained from the TriNetX database and are available from the corresponding authors upon reasonable request, subject to TriNetX data usage agreements and permissions.

## Supplementary Materials

The following supporting information can be downloaded at https://www.mdpi.com/article/10.3390/cancers17010078/s1, Table S1: Inclusion criteria; Table S2: Exclusion criteria; Table S3: ICD-10-CM codes for the outcomes.

## References

[1] Wang J.Y., Wang Q.W., Yang X.Y., Yang W., Li D.R., Jin J.Y., Zhang H.C., Zhang X.F. (2023). GLP-1 receptor agonists for the treatment of obesity: Role as a promising approach. Front. Endocrinol..

[2] Mariam Z., Niazi S.K. (2024). Glucagon-like peptide agonists: A prospective review. Endocrinol. Diabetes Metab..

[3] Alexiadou K., Hartley A., Tan T.M.M., Khamis R. (2024). The cardiovascular effects of GLP-1 receptor agonists beyond obesity and type 2 diabetes: An anti-atherosclerotic action. Trends Cardiovasc. Med..

[4] Gourgari E., Wilhelm E.E., Hassanzadeh H., Aroda V.R., Shoulson I. (2017). A comprehensive review of the FDA-approved labels of diabetes drugs: Indications, safety, and emerging cardiovascular safety data. J. Diabetes Complicat..

[5] Aslam B., Bin Zafar M.D., Changez M.I.K., Abdullah M., Safwan M., Qamar B., Shinwari A., Rai S. (2023). Exploring the potential impact of GLP-1 receptor agonists in cancer therapy. Minerva Endocrinol..

[6] Aminian A., Wilson R., Al-Kurd A., Tu C., Milinovich A., Kroh M., Rosenthal R.J., Brethauer S.A., Schauer P.R., Kattan M.W. (2022). Association of Bariatric Surgery With Cancer Risk and Mortality in Adults With Obesity. JAMA.

[7] Duca F.A., Waise T.M.Z., Peppler W.T., Lam T.K.T. (2021). The metabolic impact of small intestinal nutrient sensing. Nat. Commun..

[8] Ibrahim S.S., Ibrahim R.S., Arabi B., Brockmueller A., Shakibaei M., Büsselberg D. (2024). The effect of GLP-1R agonists on the medical triad of obesity, diabetes, and cancer. Cancer Metastasis Rev..

[9] Pyke C., Heller R.S., Kirk R.K., Ørskov C., Reedtz-Runge S., Kaastrup P., Hvelplund A., Bardram L., Calatayud D., Knudsen L.B. (2014). GLP-1 Receptor Localization in Monkey and Human Tissue: Novel Distribution Revealed With Extensively Validated Monoclonal Antibody. Endocrinology.

[10] Nauck M.A., Quast D.R., Wefers J., Pfeiffer A.F.H. (2021). The evolving story of incretins (GIP and GLP-1) in metabolic and cardiovascular disease: A pathophysiological update. Diabetes Obes. Metab..

[11] Arillotta D., Floresta G., Papanti Pelletier G.D., Guirguis A., Corkery J.M., Martinotti G., Schifano F. (2024). Exploring the Potential Impact of GLP-1 Receptor Agonists on Substance Use, Compulsive Behavior, and Libido: Insights from Social Media Using a Mixed-Methods Approach. Brain Sci..

[12] Vangoitsenhoven R., Mathieu C., Van der Schueren B. (2012). GLP1 and cancer: Friend or foe?. Endocr. Relat. Cancer.

[13] Pati S., Irfan W., Jameel A., Ahmed S., Shahid R.K. (2023). Obesity and Cancer: A Current Overview of Epidemiology, Pathogenesis, Outcomes, and Management. Cancers.

[14] Lin Y., Xu G., Li L., Xiang J., Zhai L. (2024). Incretin-based drugs decrease the incidence of prostate cancer in type 2 diabetics: A pooling-up analysis. Medicine.

[15] Piccoli G.F., Mesquita L.A., Stein C., Aziz M., Zoldan M., Degobi N.A.H., Spiazzi B.F., Lopes Junior G.L., Colpani V., Gerchman F. (2021). Do GLP-1 Receptor Agonists Increase the Risk of Breast Cancer? A Systematic Review and Meta-analysis. J. Clin. Endocrinol. Metab..

[16] Silverii G.A., Monami M., Gallo M., Ragni A., Prattichizzo F., Renzelli V., Ceriello A., Mannucci E. (2024). Glucagon-like peptide-1 receptor agonists and risk of thyroid cancer: A systematic review and meta-analysis of randomized controlled trials. Diabetes Obes. Metab..

[17] Yang Z., Lv Y., Yu M., Mei M., Xiang L., Zhao S., Li R. (2022). GLP-1 receptor agonist-associated tumor adverse events: A real-world study from 2004 to 2021 based on FAERS. Front. Pharmacol..

[18] Pasternak B., Wintzell V., Hviid A., Eliasson B., Gudbjörnsdottir S., Jonasson C., Hveem K., Svanström H., Melbye M., Ueda P. (2024). Glucagon-like peptide 1 receptor agonist use and risk of thyroid cancer: Scandinavian cohort study. BMJ.

[19] Palchuk M.B., London J.W., Perez-Rey D., Drebert Z.J., Winer-Jones J.P., Thompson C.N., Esposito J., Claerhout B. (2023). A global federated real-world data and analytics platform for research. JAMIA Open.

[20] Hu W., Song R., Cheng R., Liu C., Guo R., Tang W., Zhang J., Zhao Q., Li X., Liu J. (2022). Use of GLP-1 Receptor Agonists and Occurrence of Thyroid Disorders: A Meta-Analysis of Randomized Controlled Trials. Front. Endocrinol..

[21] Mao D., Cao H., Shi M., Wang C.C., Kwong J., Li J.J.X., Hou Y., Ming X., Lee H.M., Tian X.Y. (2021). Increased co-expression of PSMA2 and GLP-1 receptor in cervical cancer models in type 2 diabetes attenuated by Exendin-4: A translational case-control study. EBioMedicine.

[22] He L., Wang J., Ping F., Yang N., Huang J., Li Y., Xu L., Li W., Zhang H. (2022). Association of Glucagon-Like Peptide-1 Receptor Agonist Use With Risk of Gallbladder and Biliary Diseases: A Systematic Review and Meta-analysis of Randomized Clinical Trials. JAMA Intern. Med..

[23] Poommarapan K., Rummaneethorn P., Srisubat A., Suwanpidokkul N., Leenutaphong P., Nararatwanchai T., Srihirun S., Phetchengkao W., Suriyachan K., Tancharoen S. (2023). Gene Profiling of Cannabis-sativa-mediated Apoptosis in Human Melanoma Cells. Anticancer Res..

[24] Sachan N., Sharma V., Mutsuddi M., Mukherjee A. (2024). Notch signalling: Multifaceted role in development and disease. FEBS J..

[25] Liu R., Zhao E., Yu H., Yuan C., Abbas M.N., Cui H. (2023). Methylation across the central dogma in health and diseases: New therapeutic strategies. Signal Transduct. Target. Ther..

[26] Yang J.L., Chen W.Y., Chen Y.P., Kuo C.Y., Chen S.D. (2016). Activation of GLP-1 Receptor Enhances Neuronal Base Excision Repair via PI3K-AKT-Induced Expression of Apurinic/Apyrimidinic Endonuclease 1. Theranostics.

[27] Mahapatra M.K., Karuppasamy M., Sahoo B.M. (2022). Semaglutide, a glucagon like peptide-1 receptor agonist with cardiovascular benefits for management of type 2 diabetes. Rev. Endocr. Metab. Disord..

[28] Tamayo-Trujillo R., Ruiz-Pozo V.A., Cadena-Ullauri S., Guevara-Ramírez P., Paz-Cruz E., Zambrano-Villacres R., Simancas-Racines D., Zambrano A.K. (2024). Molecular mechanisms of semaglutide and liraglutide as a therapeutic option for obesity. Front. Nutr..

[29] Yaribeygi H., Maleki M., Jamialahmadi T., Sahebkar A. (2024). Anti-inflammatory benefits of semaglutide: State of the art. J. Clin. Transl. Endocrinol..

[30] Hu E.-H., Tsai M.-L., Lin Y., Chou T.-S., Chen T.-H. (2024). A Review and Meta-Analysis of the Safety and Efficacy of Using Glucagon-like Peptide-1 Receptor Agonists. Medicina.

[31] Vilsbøll T., Christensen M., Junker A.E., Knop F.K., Gluud L.L. (2012). Effects of glucagon-like peptide-1 receptor agonists on weight loss: Systematic review and meta-analyses of randomised controlled trials. BMJ.

[32] Bezin J., Gouverneur A., Pénichon M., Mathieu C., Garrel R., Hillaire-Buys D., Pariente A., Faillie J.L. (2023). GLP-1 Receptor Agonists and the Risk of Thyroid Cancer. Diabetes Care.

[33] Ryder R.E. (2013). The potential risks of pancreatitis and pancreatic cancer with GLP-1-based therapies are far outweighed by the proven and potential (cardiovascular) benefits. Diabet. Med..

[34] Liu Y., Zhang X., Chai S., Zhao X., Ji L. (2019). Risk of Malignant Neoplasia with Glucagon-Like Peptide-1 Receptor Agonist Treatment in Patients with Type 2 Diabetes: A Meta-Analysis. J. Diabetes Res..

[35] Kugler A.J., Thiman M.L. (2018). Efficacy and safety profile of once-weekly dulaglutide in type 2 diabetes: A report on the emerging new data. Diabetes Metab. Syndr. Obes..

[36] Rentzeperi E., Pegiou S., Koufakis T., Grammatiki M., Kotsa K. (2022). Sex Differences in Response to Treatment with Glucagon-like Peptide 1 Receptor Agonists: Opportunities for a Tailored Approach to Diabetes and Obesity Care. J. Pers. Med..

[37] Liu G., Li Y., Zhang T., Li M., Li S., He Q., Liu S., Xu M., Xiao T., Shao Z. (2021). Single-cell RNA Sequencing Reveals Sexually Dimorphic Transcriptome and Type 2 Diabetes Genes in Mouse Islet β Cells. Genom. Proteom. Bioinform..

[38] Michałowska J., Miller-Kasprzak E., Bogdański P. (2021). Incretin Hormones in Obesity and Related Cardiometabolic Disorders: The Clinical Perspective. Nutrients.

[39] Tan Q., Akindehin S.E., Orsso C.E., Waldner R.C., DiMarchi R.D., Müller T.D., Haqq A.M. (2022). Recent Advances in Incretin-Based Pharmacotherapies for the Treatment of Obesity and Diabetes. Front. Endocrinol..

