# Supplementary material for: Differential Effects of GLP-1 Receptor Agonists on Cancer Risk in Obesity: A Nationwide Analysis of 1.1 Million Patients
Source: Cancers (Basel). 2024 Dec 30;17(1):78. doi: 10.3390/cancers17010078 (PMC11720624; doi:10.3390/cancers17010078)
Supplement: Supplementary file 1 [file cancers-17-00078-s001.zip › cancers-3345519-supplementary.pdf]

Table S1. Inclusion criteria.

Criteria	Category	Code	Description
Known demographics	Demographics	UMLS:HL7V3.0:Race:1002-5	American Indian or Alaska Native
	Demographics	UMLS:HL7V3.0:Race:2028-9	Asian
	Demographics	UMLS:HL7V3.0:Race:2054-5	Black or African American
	Demographics	UMLS:HL7V3.0:Race:2076-8	Native Hawaiian or Other Pacific Islander
Obesity	Diagnosis	UMLS:ICD10CM:Z68.3	Body mass index 30–39, adult
	Diagnosis	UMLS:ICD10CM:Z68.4	Body mass index 40 or greater, adult
	Diagnosis	UMLS:ICD10CM:E66.0	Obesity due to excess calories (at least 18 years old at event)
GLP1R agonists	Medication	NLM:RXNORM:1991302	Semaglutide (at least 18 years at event)
	Medication	NLM:RXNORM:475968	Liraglutide (at least 18 years at event)
	Medication	NLM:RXNORM:1551291	Dulaglutide (at least 18 years at event)

Table S2. Exclusion criteria.

Criteria	Category	Code	Description
Neoplasm	Diagnosis	UMLS:ICD10CM:C00-D49	Neoplasms
Other weight loss medications	Medication	NLM:RXNORM:1440051	Lixisenatide
	Medication	NLM:RXNORM:1534763	Albiglutide
	Medication	NLM:RXNORM:60548	Exenatide
Bariatric surgery	Procedure	UMLS:CPT:1007392	Other procedures on the stomach
	Procedure	UMLS:CPT:1007385	Bariatric surgery procedures
	Diagnosis	UMLS:ICD10CM:Z98.84	Bariatric surgery status
Pregnancy	Diagnosis	UMLS:ICD10CM:Z33	Pregnant state

Table S3. ICD-10-CM codes utilized for study outcomes.

Code	Definition
C00-D49	Neoplasms
C15-C26	Malignant neoplasms of digestive organs
C15	Malignant neoplasm of esophagus
C16	Malignant neoplasm of stomach
C17	Malignant neoplasm of small intestine
C18	Malignant neoplasm of colon
C19	Malignant neoplasm of rectosigmoid junction
C20	Malignant neoplasm of rectum
C21	Malignant neoplasm of anus and anal canal
154	Malignant neoplasm of rectum, rectosigmoid junction, and anus
C25	Malignant neoplasm of pancreas
C22	Malignant neoplasm of liver and intrahepatic bile ducts
C23	Malignant neoplasm of gallbladder
C24	Malignant neoplasm of other and unspecified parts of biliary tract
C43–C44	Melanoma and other malignant neoplasms of skin
C43	Malignant melanoma of skin
C50	Malignant neoplasms of breast
C51–C58	Malignant neoplasms of female genital organs
C51	Malignant neoplasm of vulva
C52	Malignant neoplasm of vagina
C53	Malignant neoplasm of cervix uteri

C54	Malignant neoplasm of corpus uteri
C55	Malignant neoplasm of uterus, part unspecified
C56	Malignant neoplasm of ovary
C60–C63	Malignant neoplasms of male genital organs
C60	Malignant neoplasm of penis
C61	Malignant neoplasm of prostate
C62	Malignant neoplasm of testis
C64–C68	Malignant neoplasms of urinary tract
C69–C72	Malignant neoplasms of eye, brain, and other parts of central nervous system
C00–C14	Malignant neoplasms of lip, oral cavity, and pharynx
C73–C75	Malignant neoplasms of thyroid and other endocrine glands
C30–C39	Malignant neoplasms of respiratory and intrathoracic organs
C45–C49	Malignant neoplasms of mesothelial and soft tissue
C40–C41	Malignant neoplasms of bone and articular cartilage
C7A	Malignant neuroendocrine tumors
C81–C96	Malignant neoplasms of lymphoid, hematopoietic, and related tissue
